# Risk factors for mis-triage between medical and surgical causes in emergency patients with non-traumatic abdominal pain: a retrospective study

**DOI:** 10.3389/fpubh.2026.1857815

**Published:** 2026-06-22

**Authors:** Jinlong Zheng, Yang Liu, Wen Chen, Minzhu Chen, Yanhua Yin

**Affiliations:** 1 Department of Emergency, Xiangyang Central Hospital, Affiliated Hospital of Hubei University of Arts and Science, Xiangyang, China; 2 Department of Nursing, Xiangyang Central Hospital, Affiliated Hospital of Hubei University of Arts and Science, Xiangyang, China

**Keywords:** emergency department, influencing factors, non-traumatic abdominal pain, retrospective study, triage

## Abstract

**Objective:**

To analyze the current status of mis-triage between medical and surgical causes in emergency patients with non-traumatic abdominal pain, explore its influencing factors, and provide evidence-based support for optimizing emergency triage processes and improving triage accuracy.

**Design:**

This was a retrospective case-control study using data from the hospital information system and is reported in accordance with the STROBE guidelines.

**Methods:**

A retrospective case-control study was conducted. Clinical data were obtained from electronic medical records of non-traumatic abdominal pain patients who presented to the emergency department of a tertiary hospital between January 2024 and December 2025. Based on consistency between the final diagnosis and the initial triage direction (medical vs. surgical), patients were assigned to either a correctly triaged group or a mis-triage group (including medical diseases misdirected to surgery and surgical diseases misdirected to medicine). Multivariate logistic regression was performed to identify factors independently associated with mis-triage.

**Results:**

A total of 13,416 patients with non-traumatic abdominal pain were included, among whom 1,076 cases (8.02%) experienced mis-triage between medical and surgical causes. Of these, 58.10% were surgical diseases misdirected to medicine, and 41.90% were medical diseases misdirected to surgery. Multivariate logistic regression identified the following factors independently associated with mis-triage: number of accompanying symptoms (*OR* = 1.224, *95%CI*: 1.094–1.370), pain score (*OR* = 0.783, *95%CI:* 0.741–0.828), working years of triage nurse (*OR* = 0.960, *95%CI*: 0.944–0.977), mode of arrival (*OR* = 0.959, *95%CI*: 0.942–0.975), male sex *(OR* = 1.140, *95%CI*: 1.005–1.293), and walking (*OR* = 1.757, *95% CI*: 1.301–2.371) (all *P* < 0.05).

**Conclusion:**

A certain proportion of mis-triage occurs in emergency patients with non-traumatic abdominal pain, with surgical diseases being more frequently misdirected to medicine. A higher number of accompanying symptoms, lower pain score, triage by less experienced nurses, and presence of fever are major risk factors for triage error. It is recommended to strengthen specialized training for emergency triage nurses, develop triage support tools integrating typical symptoms with point-of-care testing, and enhance dynamic assessment for older adults with more accompanying symptoms, in order to reduce mis-triage rates and ensure patient safety.

## 1 Introduction

Acute non-traumatic abdominal pain is one of the most common chief complaints in emergency departments, accounting for 5–20% of all emergency visits (1). Its etiologies involve multiple systems, including the digestive, urinary, reproductive, and cardiovascular systems, ranging from gastroenteritis to highly lethal conditions such as acute myocardial infarction, aortic dissection, and mesenteric ischemia. Consequently, the differential diagnosis is extremely complex and encompasses a wide range of medical and surgical specialties (2).

As the first critical step in risk assessment of emergency patients, triage accuracy directly affects the timing of treatment initiation and ultimately patient outcomes (3). However, due to the constraints of limited triage time, incomplete information, and atypical presentations of some diseases, the rate of triage errors for non-traumatic abdominal pain remains high. A retrospective analysis of 860 patients by Wang et al. (4). reported a triage misdiagnosis rate of 5.2%, with five major contributing factors: involvement of multiple intersecting specialties, different diseases presenting with identical symptoms, concealment of medical history by patients or families, nurse-related factors, and increased patient volume in the emergency department (4).

Another observational study investigating 1,747 patients who called emergency medical services for abdominal pain found that 12.8% were triaged to home self-care, of whom 16.3% revisited within 96 h for the same symptoms, and 5.6% required hospitalization (5). Furthermore, six patients were retrospectively judged to have received inappropriate prehospital triage, with final diagnoses including ruptured abdominal aortic aneurysm, acute appendicitis with peritonitis, and acute pancreatitis-all time-sensitive emergencies (5). These findings indicate that triage evaluation for abdominal pain poses significant decision-making challenges in both prehospital and in-hospital settings.

Triage errors not only lead to inappropriate patient referral (e.g., misdirecting a myocardial infarction to a gastroenterology department), thereby delaying specific treatment, but may also prolong waiting time due to underestimation of triage level and even trigger medical disputes (6). Non-traumatic acute abdominal pain often presents with rapid onset, swift progression, and potential severity. Without timely diagnosis and management, patients may experience clinical deterioration or even death within a short period. Emergency triage is therefore a critical step in risk assessment for this patient population (7). Consequently, identifying modifiable risk factors for triage errors is of great importance for optimizing triage workflows and ensuring patient safety.

Previous studies have primarily focused on evaluating the reliability and validity of triage instruments [e.g., ESI (8), CTAS (9, 10)] or exploring misdiagnosis of abdominal pain due to reproductive system diseases in female patients (11, 12). However, sex differences and biases in triage decision-making are receiving increasing attention. Using a large language model to analyze real-world emergency triage data from Bordeaux University Hospital in France, Dorémus et al. (13) found significant sex bias among triage nurses: under identical clinical conditions, female patients were assigned lower triage severity levels than male patients, and this bias was more pronounced among female nurses. Meanwhile, in a retrospective analysis of patients with acute abdominal pain, Hayes et al. (14) observed sex differences in pain management: although male patients were more likely to receive a combination of opioid and non-opioid analgesics as first-line pain relief, female patients had a significantly higher proportion of receiving first analgesia more than 90 min after emergency department arrival, suggesting sex differences unrelated to clinical condition in the assessment and management of acute abdominal pain. Moreover, a study on factors associated with missed diagnosis of aortic dissection in the emergency department identified absence of severe pain, non-urgent triage category, and failure to use point-of-care ultrasound as independent factors linked to missed diagnosis (14, 15).

In summary, although preliminary investigations into triage assessment for non-traumatic abdominal pain have been conducted by researchers worldwide, systematic evidence based on multivariate regression analysis remains lacking regarding several key issues. These include the independent role of male sex in triage risk, the bidirectional influence of the number of accompanying symptoms, the triage weighting of mode of arrival, and the protective effect of triage nurse experience. Therefore, this study aimed to describe the prevalence of mis-triage between medical and surgical causes in emergency patients with non-traumatic abdominal pain, identify the factors independently associated with it, and provide empirical evidence for developing multidimensional triage warning strategies.

## 2 Methods

### 2.1 Participants

**Figure 1:**
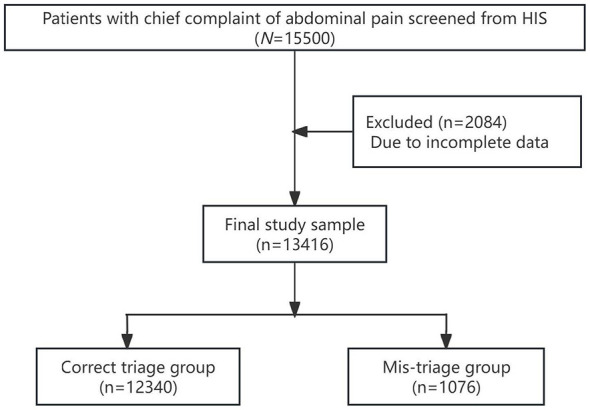
Study flow diagram of participant selection and group allocation.

This was a retrospective case-control study using data from the hospital information system. We consecutively collected medical records of patients who presented to the emergency department of our hospital with non-traumatic abdominal pain (NTAP) between January 2024 and December 2025 from the hospital information system (HIS). Inclusion criteria were: (1) chief complaint of abdominal pain at emergency admission; (2) age >18 years; (3) complete medical records with a definite discharge diagnosis or documented disposition to a specialty inpatient department. Exclusion criteria were: (1) abdominal pain resulting from definite trauma such as a traffic accident or fall; (2) pregnant or postpartum patients; (3) patients who had already received a definitive diagnosis at another hospital and were transferred for further treatment; (4) patients presenting with cardiac or respiratory arrest requiring immediate resuscitation. The study was approved by the Ethics Committee of Xiangyang Central Hospital (approval No. 20251-187), and all participants provided informed consent. The sample selection process is shown in Figure 1.

### 2.2 Outcome measures

Definition of outcome variables: triage was considered correct when the initial triage assignment (medical or surgical) was consistent with the final diagnostic department. Triage was considered incorrect (mis-triage) when the initial triage assignment was inconsistent with the final diagnostic department (e.g., triaged to internal medicine but ultimately treated surgically or transferred to a surgical ward).

Definitions of medical vs. surgical abdominal pain: medical abdominal pain was defined as conditions primarily managed by internal medicine departments, including but not limited to gastroenteritis, acute pancreatitis, biliary colic without surgical intervention, and non-surgical renal colic. Surgical abdominal pain was defined as conditions requiring surgical evaluation or intervention, including acute appendicitis, cholecystitis, intestinal obstruction, perforated viscus, and abdominal vascular emergencies (e.g., acute mesenteric ischemia, ruptured abdominal aortic aneurysm). The final diagnosis was determined by the attending physician at discharge or upon transfer to a specialty ward.

1 General data collection form

Demographic characteristics: sex, age, chief complaint, accompanying symptoms (fever, vomiting, diarrhea, hematuria, etc.,), and past medical history (history of surgery, diabetes, hypertension, etc.,).

2 Disease-related data collection form

This included immediate triage vital signs: temperature (T), heart rate (HR), respiratory rate (R), blood pressure (BP), peripheral oxygen saturation (SpO_2_), pain score, Modified Early Warning Score (MEWS), and Emergency Severity Index (ESI) level. Vital signs were measured by triage nurses using Mindray patient monitors.

3 Numerical Rating Scale (NRS) for pain

The NRS is one of the most widely used clinical pain assessment tools. It consists of an 11-point scale from 0 to 10, where 0 represents “no pain” and 10 represents “the most intense pain imaginable.” Patients select a number that best reflects their pain intensity (16). Pain assessment was performed by triage nurses through patient interviews.

4 Emergency Severity Index (ESI)

The ESI was jointly developed by an American team of emergency physicians and nurses in the late 1990's, subsequently refined with funding from the Agency for Healthcare Research and Quality (AHRQ), and progressively optimized through clinical practice. Based on two core dimensions—patient acuity and expected resource utilization—the ESI classifies emergency patients into five levels, from level 1 (most urgent) to level 5 (least urgent), enabling rapid categorization and prioritization (8).

5 Modified Early Warning Score (MEWS)

The MEWS is a rapid physiological assessment tool proposed by the UK's “risk patient emergency response team” in the 1990's. It quantifies five physiological parameters: heart rate, systolic blood pressure, respiratory rate, temperature, and level of consciousness. The total score ranges from 0 to 14, with higher scores indicating greater illness severity. MEWS was calculated by triage nurses based on vital signs obtained at the time of triage (17).

### 2.3 Data collection

Data extraction was performed independently by two trained nursing postgraduate students. The extraction procedure was as follows:

1. Emergency visit records with the chief complaint of “abdominal pain” or “acute abdominal pain” were retrieved from the hospital information system (HIS).
2. The inclusion and exclusion criteria were applied sequentially to determine the final study sample.
3. Using a pre-designed structured data collection form, the following information was extracted from electronic medical records: patient demographics (age, sex, time of arrival, mode of arrival), triage records (pain score, accompanying symptoms, triage nurse identifier, triage assignment, ESI level), emergency course (vital signs, initial diagnosis, disposition), and final diagnosis (discharge diagnosis).
4. Data extraction was performed independently by two researchers using a standardized data collection form. After extraction, the two datasets were cross-checked by a third researcher, and any discrepancies were resolved by reviewing the original electronic medical records to ensure data accuracy.

### 2.4 Statistical analysis

Statistical analyses were performed using SPSS 26.0. Continuous variables with a normal distribution were presented as mean ± standard deviation, and comparisons between groups were conducted using the independent samples *t*-test. Non-normally distributed continuous variables were presented as median (interquartile range, IQR) and compared using the Mann–Whitney *U*-test. Categorical variables were expressed as frequencies and percentages and compared using the chi-square (χ^2^) test. Variables with a *P* < 0.05 in univariate analysis were entered as candidates into a multivariate logistic regression model to identify factors independently associated with mis-triage between medical and surgical causes. Additionally, variance inflation factor (VIF) was calculated to check for multicollinearity among independent variables (VIF < 5 considered acceptable). A two-sided α of 0.05 was used as the threshold for statistical significance, with *P* < 0.05 considered statistically significant.

## 3 Results

### 3.1 General participants characteristics

A total of 15,500 patients were initially enrolled in this study. After excluding cases with incomplete data, 13,416 patients were finally included. The age ranged from 18 to 104 years, with a median of 29 (*IQR:* 29, 62). There were 6,495 males (48.41%) and 6,921 females (51.59%). The most common mode of arrival was walking (84.19%), followed by transfer from another hospital (8.74%), ambulance (3.64%), and wheelchair (3.42%). A history of hypertension was present in 22.30% of patients, fever was noted in 2.28%, and 21.29% presented with one or more accompanying symptoms.

### 3.2 Univariate analysis of factors associated with mis-triage between medical and surgical causes in emergency patients with non-traumatic abdominal pain

A total of 1,076 cases (8.02%) of mis-triage between medical and surgical causes were identified, including 283 cases of medical diseases misdirected to surgery and 793 cases of surgical diseases misdirected to medicine. Univariate analysis showed that patient age, sex, number of accompanying symptoms, presence of fever, pain score, history of hypertension, MEWS score, ESI level, and working years of the triage nurse were significantly associated with triage errors (all *P* < 0.05), as shown in Table 1.

**Table 1:** Univariate analysis of factors associated with mis-triage between medical and surgical causes in emergency patients with non-traumatic abdominal pain.

Variable	Category	Total	Correct triage group (*n* = 12,340)	Mis-triage group (*n* = 1,076)	Test statistic *(t, Z, χ^2^)*	*P* value
Sex	Female	6,921 (51.59)	6,397 (51.84)	524 (48.70)	3.909	0.048
	Male	6,495 (48.41)	5,943 (48.16)	552 (51.30)		
Number of accompanying symptoms	0	10,560 (78.71)	9,783 (79.28)	777 (72.21)	31.838	0.000
	1	2,378 (17.73)	2,128 (17.24)	250 (23.23)		
	2	457 (3.41)	412 (3.34)	45 (4.18)		
	3	21 (0.16)	17 (0.14)	4 (0.37)		
Mode of arrival	Ambulance	489 (3.64)	463 (3.75)	26 (2.42)	24.080	0.000
	Transfer from another hospital	1,173 (8.74)	1,114 (9.03)	59 (5.48)		
	Walking	11,295 (84.19)	10,334 (83.74)	961 (89.31)		
	Wheelchair	459 (3.42)	429 (3.48)	30 (2.79)		
Pain location	Upper abdomen	8,217 (61.25)	7,562 (61.28)	655 (69.87)	0.069	0.793
	Lower abdomen	5,199 (3)	4,778 (38.72)	421 (39.13)		
ESI Levels	Level 1	94 (0.70)	83 (0.67)	11 (1.02)	18.186	0.000
	Level 2	1,693 (12.62)	1,600 (12.97)	93 (8.64)		
	Level 3	11,629 (86.68)	10,657 (86.36)	972 (90.33)		
MEWS Levels	Level 1	57 (0.42)	51 (0.41)	6 (0.56)	0.936	0.817
	Level 2	273 (2.03)	254 (2.06)	19 (1.77)		
	Level 3	2,205 (16.44)	2,030 (16.45)	175 (16.26)		
	Level 4	10,881 (81.10)	10,005 (81.08)	876 (81.41)		
Hypertension	No	10,424 (77.70)	9,618 (77.94)	806 (74.91)	5.260	0.022
	Yes	2,992 (22.30)	2,722 (22.06)	270 (25.09)		
Presence of Fever	No	13,110 (97.72)	12,047 (97.63)	1,063 (98.79)	6.039	0.014
	Yes	306 (2.28)	293 (2.37)	13 (1.21)		
Age (years) *(M_25_, M_75_)*			46.000 (29.0,62.0)	50.0 (32.0,64.0)	−3.357	0.001^**^
Mews Scores (*M_25_, M_75_*)			1.00 (0.0,1.0)	1.0 (0.0,1.0)	−1.983	0.047^*^
NRS Scores (*M_25_, M_75_*)			3.00 (3.0,4.0)	3.0 (2.0,4.0)	−8.858	0.000^**^
Working years of triage nurses (*M_25_, M_75_*)			10.0 (7.0,15.0)	10.0 (7.0,12.0)	−4.279	0.000^**^

Data are presented as *n* (%) for categorical variables and as median (*M*_2_*5, M75*) for continuous variables, where *M*_2_*5* and *M75* represent the 25th and 75th percentiles, respectively. *OR*, odds ratio; *CI*, confidence interval; ESI, Emergency Severity Index; MEWS, Modified Early Warning Score; NRS, Numerical Rating Scale. ^*^P < 0.05, ^**^P < 0.001.

### 3.3 Multivariate analysis of factors associated with mis-triage between medical and surgical causes in emergency patients with non-traumatic abdominal pain

Collinearity diagnostics were performed on the independent variables that were statistically significant in the univariate analysis. The variance inflation factor (VIF) for all variables was < 5, indicating no significant multicollinearity. A binary logistic regression analysis was conducted with triage accuracy as the dependent variable and the statistically significant variables from the univariate analysis as independent variables. The multivariate logistic regression results showed that patient sex, age, mode of arrival, number of accompanying symptoms, NRS pain score, and working years of the triage nurse were independent factors influencing triage accuracy. The model explained 64.25% of the total variance (Table 2).

**Table 2:** Multivariate logistic regression analysis of triage error in emergency patients with non-traumatic abdominal pain.

Variables	*B*	*SE*	*Wald χ2*	*P*	*OR*	***95% CI***	
						*Lower*	*Upper*
Constant	−1.171	0.410	8.157	0.004	0.310		
Sex (1)	0.131	0.064	4.128	0.042	1.140	1.005	1.293
Age	0.007	0.002	15.649	0.000	1.007	1.003	1.010
Number of accompanying symptoms	0.202	0.057	12.419	0.000	1.224	1.094	1.370
NRS Scores	−0.245	0.028	74.672	0.000	0.783	0.741	0.828
ESILevels			8.927	0.012			
Level 2	−1.074	0.361	8.841	0.003	0.341	0.168	0.693
Level 3	−0.976	0.367	7.090	0.008	0.377	0.184	0.773
Mode of arrivals			14.630	0.002			
Walking	0.563	0.153	13.548	0.000	1.757	1.301	2.371
Working years of triage nurses	−0.042	0.009	23.399	0.000	0.959	0.942	0.975

Reference categories-Sex: female; ESI level: level I; Mode of arrival: transfer from another hospital. All continuous variables (age, number of accompanying symptoms, NRS pain score, working years of triage nurse) were entered with their original values. *OR*, odds ratio; *CI*, confidence interval.

## 4 Discussion

### 4.1 General patient characteristics and mis-triage rate

In this study, the overall mis-triage rate for patients with non-traumatic abdominal pain was 8.02%, which is slightly lower than the 16.3% reported in another study (18). This difference may be attributed to the fact that the present study only included patients with non-traumatic abdominal pain. Although the accuracy was somewhat higher than that in previous reports, the current mis-triage rate remains substantially high. In particular, missed diagnoses of low-probability but high-risk life-threatening diseases (e.g., acute myocardial infarction, aortic dissection) pose a serious threat to patient outcomes. Among the mis-triage cases, 58.1% were surgical diseases misdirected to medicine, and 41.9% were medical diseases misdirected to surgery. The majority of patients arrived by walking (84.19%), and the median age was 29 years (IQR: 29, 62). These findings highlight the urgency and necessity of systematically identifying factors influencing triage errors and developing multidimensional triage warning strategies.

### 4.2 Factors associated with mis-triage in univariate analysis

Univariate analysis identified several variables significantly associated with triage error, including presence of fever, history of hypertension, ESI level, and MEWS score (all *P* < 0.05, see Table 1). However, these factors did not remain statistically significant in the multivariate model. The lack of independent association may be explained by confounding with other stronger predictors (e.g., age, number of accompanying symptoms) or by the relatively low prevalence of fever (2.28%) and hypertension (22.30%) in our cohort. For instance, fever was actually less frequent in the mis-triage group (1.21% vs. 2.37% in the correct triage group), possibly because many surgical conditions (e.g., early appendicitis) may not present with fever, and febrile patients tend to receive higher triage vigilance. ESI level 2 showed a protective effect in univariate analysis (*OR* = 0.439, *95%CI*: 0.229–0.840), but this did not persist after adjusting for other variables, suggesting that the urgency level assigned by triage nurses is itself influenced by the same clinical features (pain score, vital signs, etc.,) that we included in the multivariate model. These univariate findings are informative for hypothesis generation but should be interpreted with caution.

### 4.3 Factors independently associated with mis-triage in multivariate analysis

#### 4.3.1 Sex

Male sex (*OR* = 1.140, 95%CI: 1.005–1.293, *P* = 0.042) was an independent factor associated with mis-triage, consistent with Li et al. (19). At the clinical level, men are at higher risk of coronary artery disease, and both inferior wall myocardial infarction and aortic dissection often present with abdominal pain as the predominant symptom (abdominal type myocardial infarction and abdominal type aortic dissection). Moreover, the well-developed greater omentum in men may temporarily mask signs of peritonitis, leading to atypical early abdominal presentations and thus increasing triage difficulty (20). At the behavioral level, traditional masculine culture tends to encourage men to delay seeking medical care, underestimate pain intensity, and use vague language to describe symptoms, thereby reducing the quality of chief complaint information on which triage systems rely, and systematically underestimating triage levels (21). At the cognitive and systemic level, triage nurses may have a mindset that “abdominal pain in men is commonly due to medical or surgical diseases,” lacking vigilance for atypical presentations (9). Meanwhile, existing triage tools (e.g., ESI, MEWS) do not incorporate sex-related differences in physiological compensation. Men's better cardiopulmonary reserve allows vital signs to remain within normal range even in early shock, further masking the severity of illness. These three sets of factors interact and reinforce each other, forming a “risk triangle” for triage errors in male patients.

#### 4.3.2 Age

Age (*OR* = 1.007, *95% CI*: 1.003–1.010, *P* < 0.001) was independently associated with mis-triage. Each 1-year increase in age was associated with a 0.7% increase in the risk of triage error. Although this effect size appears small, the cumulative effect of a continuous variable leads to a significantly elevated risk in older patients. Older adults often have multiple comorbid conditions, elevated pain thresholds, and blunted peritoneal irritation responses. Consequently, the degree of abdominal pain often does not match the pathological severity, and the disease presentation is frequently atypical, leading to delayed medical care, increased clinical complexity, and greater illness severity. These factors interfere with triage nurses' clinical judgment and increase the risk of triage errors (10).

#### 4.3.3 Number of accompanying symptoms

The number of accompanying symptoms (*OR* = 1.224, *95% CI*: 1.094–1.370, *P* < 0.001) was independently associated with mis-triage. Each additional accompanying symptom increased the odds of triage error by 22.4%. Accompanying symptoms serve both as warning signals and as major pitfalls that can mislead triage decisions. Their effect is bidirectional: too few symptoms (e.g., isolated abdominal pain) may lead to oversimplification, causing nurses to overlook life-threatening emergencies such as abdominal type myocardial infarction or aortic dissection; too many symptoms may overlap with common medical diseases (e.g., acute gastroenteritis), leading to cognitive shortcuts and prioritization of high-incidence conditions over low-incidence but high-mortality vascular events. Furthermore, information overload may distract nurses from recognizing core signs (e.g., radiating pain, profuse sweating), further increasing misjudgment.

#### 4.3.4 Mode of arrival

Compared with patients transferred from another hospital, those who arrived by walking had significantly higher odds of triage error (*OR* = 1.757, *95%CI:* 1.301–2.371, *P* < 0.001). Walking patients are often presumed to have “mild illness with the ability to move independently” and are consequently assigned lower triage levels (e.g., ESI level 4 or 5), even though their actual condition may be at an early or atypical stage. In contrast, patients arriving by ambulance or transfer from another hospital generally present with greater illness severity, and the mode of arrival itself triggers higher triage vigilance, paradoxically reducing mis-triage risk.

#### 4.3.5 Interaction of multiple factors

The four factors described above do not act in isolation but interact with and reinforce each other. For example, an older adult who arrives by walking and presents with isolated abdominal pain simultaneously carries risks from all four dimensions, resulting in a geometric increase in triage error risk. Based on our findings, we propose the following recommendations for triage practice (noting that these procedures were not implemented in the present retrospective study): for patients aged >65 years with abdominal pain, an electrocardiogram should be routinely performed to rule out myocardial infarction, regardless of the mode of arrival; for patients who arrive by walking or private car but present with vague chief complaints, a brief secondary assessment should be implemented; for patients with an abnormal number of accompanying symptoms, closed-ended questions should be used to systematically screen for cardiovascular and vascular-related signs; and for male patients, especially those of middle age or older, vigilance for abdominal type myocardial infarction and aortic dissection should be strengthened.

#### 4.3.6 Pain score

Pain score (*OR* = 0.783, *95%CI*: 0.741–0.828, *P* < 0.001) was identified as a protective factor-higher self-reported pain intensity was associated with lower risk of mis-triage. Pain serves as a biological warning signal, and the behavioral signs of severe pain (distressed facial expression, agitation, forced posture) provide direct evidence of illness severity. In this state of heightened vigilance, triage nurses are more likely to initiate comprehensive assessment, including detailed inquiry into accompanying symptoms, more frequent vital sign monitoring, and a lower threshold for specialty triage (11). Therefore, while patients with high pain scores may have more severe conditions, the strong “pain signal” itself paradoxically attracts more thorough triage attention, reducing missed diagnosis. Future triage optimization should preserve the protective effect of high pain scores while compensating for low-pain patients through multidimensional information integration (age, sex, accompanying symptoms, mode of arrival).

#### 4.3.7 Working years of triage nurse

The working years of the triage nurse (*OR* = 0.959, *95%CI*: 0.942–0.975, *P* < 0.001) was also a protective factor- each additional year of clinical experience was associated with an approximately 4.1% reduction in mis-triage risk. This finding is consistent with Wu et al. (12). According to Benner's novice-to-expert framework (22), with increasing experience, nurses develop better pattern recognition, more efficient multivariate information integration, and reduced susceptibility to cognitive biases. Nursing managers should stabilize the triage workforce and strengthen structured training for less experienced nurses to maximize this protective effect.

## 5 Conclusion

This study demonstrated that patient age, number of accompanying symptoms, male sex, and arrival by walking were independent risk factors for mis-triage in emergency patients with non-traumatic abdominal pain. Emergency triage is the first step in the emergency care process and plays a critical role in alleviating emergency department crowding and identifying high-risk patients with abdominal pain. Based on our findings, we propose the following recommendations for triage practice, with the understanding that these procedures were not implemented in the present retrospective study: for patients aged >65 years, male, arriving by walking, presenting with multiple accompanying symptoms, or showing a mismatch between pain score and behavioral presentation, routine electrocardiographic and vascular disease screening should be considered; a brief secondary assessment should be performed; and closed-ended questions should be used to compensate for insufficient information provision. Furthermore, nursing managers should stabilize the triage nursing workforce and strengthen structured training for less experienced nurses to maximize the protective effect of experience accumulation, thereby systematically reducing the risk of triage errors in patients with non-traumatic abdominal pain.

## 6 Limitations and future directions

This study is a single-center retrospective case analysis, with data derived from the emergency triage records of one medical institution, which may introduce selection bias. Triage processes, patient composition, and nursing staffing levels may vary across hospitals of different tiers and geographic regions. Therefore, future multicenter, large-scale prospective studies are warranted to validate our findings. In addition, although this study included variables such as patient age, sex, number of accompanying symptoms, pain score, mode of arrival, and working years of triage nurses, it did not comprehensively capture other potentially relevant factors, including pre-hospital medication use (e.g., analgesics or beta-blockers that may mask symptoms), time of emergency department visit (e.g., night shifts or holidays when triage staffing may be relatively weaker), and the professional title, psychological status, or fatigue level of the triage nurse. These factors may independently or interactively influence triage decisions, and their omission could lead to residual confounding in the estimation of certain effects. Future research using prospective multicenter designs, standardized data collection tools, more comprehensive covariate adjustment, and qualitative studies of triage nurses' cognitive processes is needed to further validate and extend the findings of this study.

## Data Availability

The original contributions presented in the study are included in the article/supplementary material, further inquiries can be directed to the corresponding author.
